# Allogenic perinatal tissue for musculoskeletal regenerative medicine applications: a systematic review protocol

**DOI:** 10.1186/s13018-022-03197-z

**Published:** 2022-06-11

**Authors:** Adarsh Aratikatla, Nicola Maffulli, Hugo C. Rodriguez, Manu Gupta, Anish G. Potty, Saadiq F. El-Amin, Ashim Gupta

**Affiliations:** 1 The Royal College of Surgeons in Ireland, Dublin, Ireland; 2 Department of Musculoskeletal Disorders, School of Medicine and Surgery, University of Salerno, 84084 Fisciano, Italy; 3 San Giovanni di Dio e Ruggi D’Aragona Hospital “Clinica Orthopedica” Department, Hospital of Salerno, 84124 Salerno, Italy; 4 Centre for Sports and Exercise Medicine, Barts and the London School of Medicine and Dentistry, Queen Mary University of London, London, E1 4DG UK; 5 School of Pharmacy and Bioengineering, Keele University School of Medicine, Stoke-on-Trent, ST5 5BG UK; 6 Holy Cross Hospital, Orthopaedic Research Institute, Fort Lauderdale, FL 33334 USA; 7 Department of Orthopaedic Surgery, Larkin Community Hospital, South Miami, FL USA; 8 Future Biologics, Lawrenceville, GA 30043 USA; 9 Polar Aesthetics Dental & Cosmetic Centre, Noida, Uttar Pradesh 201301 India; 10 South Texas Orthopaedic Research Institute (STORI Inc.), Laredo, TX 78045 USA; 11 Laredo Sports Medicine Clinic, Laredo, TX 78041 USA; 12 El-Amin Orthopaedic & Sports Medicine Institute, Lawrenceville, GA 30043 USA; 13 Regenerative Sports Medicine, Lawrenceville, GA 30043 USA; 14 BioIntegrate Inc., 2505 Newpoint Pkwy, Suite – 100, Lawrenceville, GA 30043 USA; 15 Veterans in Pain (V.I.P.), Valencia, CA 91354 USA; 16 Indian Stem Cell Study Group (ISCSG) Association, Lucknow, Uttar Pradesh 110048 India

**Keywords:** Regenerative medicine, Musculoskeletal injuries, Umbilical cord, Wharton’s jelly, Amniotic tissue, Amniotic fluid, Amniotic membrane, Perinatal tissue, PRISMA

## Abstract

**Background:**

Musculoskeletal ailments impact the lives of millions of people, and at times necessitate surgery followed by physiotherapy, drug treatments, or immobilization. Regenerative musculoskeletal medicine has undergone enormous progress over the last few decades. Sources of tissues used for regenerative medicine purposes can be grouped into autologous or allogenic. Although autologous sources are promising, there is a wide range of limitations with the treatment, including the lack of randomized controlled studies for orthopaedic conditions, donor site morbidity, and highly variable outcomes for patients. Allogenic sources bypass some of these shortcomings and are a promising source for orthopaedic regenerative medicine applications.

**Methods:**

A systematic search will be performed using PubMed, Elsevier, ScienceDirect, and Google Scholar databases for articles published in English before May 2022. The Preferred Reporting Items for Systematic Reviews and Meta-Analyses statement and guidelines will be used. Studies will be eligible if they apply to acute and chronic orthopaedic musculoskeletal complications or animal or human disease models. Publications must include the use of MSCs and/or tissue obtained from amniotic/chorionic membrane, amniotic fluid, umbilical cord, and/or umbilical cord-derived Wharton’s jelly as an intervention. Placebos, noninjury models, acute injury models, non-injury models, and gold standard treatments will be compared. The study selection will be performed by two independent reviewers using a dedicated reference management software. Data synthesis and meta-analysis will be performed separately for preclinical and clinical studies.

**Discussion:**

The results will be published in relevant peer-reviewed scientific journals. Investigators will present results at national or international conferences.

*Trial registration*: The Protocol will be registered on PROSPERO international prospective register of systematic reviews prior to commencement.

## Background

Musculoskeletal injuries are classified as any injury that affects bones, muscles, ligaments, tendons, and nerves [1]. These ailments bring pain to millions of people each year and costs healthcare services an estimated $54 billion every year [2]. Current and conventional methods of treatment for such conditions are limited, as they often do not address the pathology that initially caused the damage [3–5]. Presently, non-surgical methods for the relief of orthopaedic injuries include lifestyle changes such as exercise, diet, physical therapy, and weight loss [6–9]. Other treatments involve non-steroidal anti-inflammatory drugs (NSAIDs), viscosupplementation, corticosteroid injections, and opioids [6, 10–12]. NSAIDs do aid the patient’s pain in the short-term, but do not address the underlying problem [13]. In viscosupplementation, hyaluronic acid is injected into a patients joint. This hyaluronic acid is supposed to act as a lubricant and reduce discomfort and facilitate movement, but many patients do not necessarily experience an improvement in their function [6]. Corticosteroid injections can also help reduce inflammation and pain [14], but they only provide interim relief [14]. Parenteral and epidural opioids are relatively effective in relieving postoperative pain, but orthopaedic surgeons have many hesitations to prescribe them [15]. For example, mild side effects for opioid medications include constipation, sedation, and vomiting, and more severe adverse effects entail hypertension, respiratory problems, urinary difficulties, and dehydration [15]. Furthermore, a major cause for concern when prescribing opioids for pain management is the potential for a patient to develop dependence on the drug [15]. While all these treatment methods are somewhat beneficial, they all seem to be temporary solutions to the patient’s underlying problem. In response to these limitations, researchers and clinicians have shown increased interest in the use of regenerative medicine-based modalities [16–19]. These regenerative medicine modalities include autologous and allogenic sources. The main types of autologous treatments include platelet-rich plasma (PRP) injections, bone marrow transplants (BMTs), and the use of adipose-derived tissues (ADTs) and/or cells (ADCs) [20–23]. Although promising, these autologous sources have limitations. For example, the efficacy of PRP injections is highly variable, as basic science research studies show that their effects can be both pro- and anti-inflammatory and the therapeutic results depend on several factors [24]. BMT, or hematopoietic stem cell transplantation, also presents its own limitations, as patients were more likely to experience post-operative complications compared to patients who underwent an allogenic BMT [25]. One of the most common means of obtaining ADT or ADCs for autologous treatment is via stromal vascular fraction (SVF). SVF is an accessible, minimally manipulated array of cells that can be used for autologous regenerative treatment [26], which can be isolated either enzymatically or mechanically [27]. Although this is a promising method, there is a dearth of randomized controlled studies for orthopaedic conditions [28]. Additionally, autologous transplants are susceptible to the relapse of tumor if the autograft contains malignant cells [29]. Autologous ADTs/ADCs and BMTs pose may carry point of harvest morbidity [30–32]. To overcome these limitations, and develop an alternate supply, clinicians have begun exploring the use of allogenic sources bypassing some of the drawbacks associated with autologous tissue/sources. The allogenic perinatal tissues discussed in this article include the amnion/chorion membrane (ACM), amniotic fluid (AF), umbilical cord (UC), umbilical cord-derived Wharton’s jelly (UC-WJ), and mesenchymal stromal/stem cells (MSCs) derived from these tissues. There is ample literature showing the safety and efficacy of autologous sources, but there is limited research concerning perinatal allogenic sources. This review aims to document the preclinical and clinical outcomes of different perinatal allogenic tissues and/or derived cells for orthopaedic regenerative medicine applications. The secondary goal is to list all ongoing clinical trials enlisted on ClinicalTrials.gov related to different perinatal allogenic tissues and/or derived cells for orthopaedic regenerative medicine applications.

## Methods

The systematic review will be registered on the PROSPERO international prospective register of systematic reviews and will follow the Preferred Reporting Items for Systematic Reviews and Meta-Analyses (PRISMA) statement and guidelines [3, 33].

### Eligibility criteria

The PICOS (Population, Intervention, Comparison, Outcome, Study Design) framework will be used as a template for defining eligibility criteria for literature search [34].

### Population

Research involving animal models and humans (preclinical and clinical studies) will be considered for review, without exclusion relating to sex or age. Preclinical studies must include human tissues and/or cells isolated from a living model. Studies meeting eligibility criteria will apply to both acute and chronic orthopaedic musculoskeletal complications or disease models. Articles will be excluded if they do not relate to orthopaedic intervention.

### Intervention

Research meeting inclusion criteria will involve the use of MSCs and/or tissue obtained from amniotic/chorionic membrane, amniotic fluid, umbilical cord, and/or umbilical cord-derived Wharton’s jelly. Studies will be excluded if amniotic/chorionic membrane, amniotic fluid, umbilical cord, and/or umbilical cord-derived Wharton’s jelly is not included in the experimental tissue and/or cell type examined. Studies will be excluded if they report the use of amniotic/chorionic membrane, amniotic fluid, umbilical cords, and/or umbilical cord-derived Wharton’s jelly tissue and/or MSCs in combination with other tissue and/or cell populations.

### Comparison

Comparators considered will include placebos, noninjury models, acute injury models, non-injury models, and gold standard treatments for orthopaedic injury.

### Outcomes

For basic scientific and/or preclinical research, studies relating to human musculoskeletal injury reporting histological or biochemical measures will be included. For clinical research, studies pertaining to human orthopaedic musculoskeletal injury reporting histological and/or biochemical measures and functional scores (activity, quality of life, pain, etc.) will be included.

### Study design

Observational studies (cohort, cross-sectional, and case-controlled prospective or retrospective studies) or randomized controlled trials comparing outcomes of tissue and/or MSCs derived from amniotic/chorionic membrane, amniotic fluid, umbilical cords, and/or umbilical cord-derived Wharton’s jelly with control, experimental therapy, or gold standard treatment regardless of their length of follow-up. Systematic reviews and meta-analyses will only be examined to identify further studies for inclusion, and results of meta-analyses will not be included in the analysis. There will be no limitation based on date of publication.

### Information sources

A systematic search will be conducted in PubMed, Elsevier, Google Scholar, and ScienceDirect databases of English language articles published before May 2022. Secondary searching of reference lists of key articles, narrative and systematic reviews, and meta-analyses will be undertaken to identify any additional studies potentially missed in electronic search.

### Search

The search and selection process will be based on the PRISMA checklist and flow diagram based on the eligibility and inclusion criteria previously outlined. A web-based reference software system (RefWorks) will be used for data management.

### Study selection

Two reviewers will independently perform the study selection process. Screening of abstracts will be performed, and full text articles will be retrieved and uploaded to the reference management software. A thorough secondary screening will be performed independently by two reviewers. The secondary screening of the full text articles will eliminate studies that do not meet inclusion criteria.

### Data collection

Data extraction from articles that meet inclusion criteria will be performed by two independent reviewers. Data extracted and synthesized will include authors, publication year, study design, group controls, group interventions, outcome measurement, and outcome assessment. Customized forms will be used in the data extraction and collection process. The primary authors will be contacted via email for any information necessitating clarification.

### Data items

Relevant items of population, problem, intervention, comparison, and outcome will be extracted and included. For basic scientific research, relevant histological and/or biochemical measures will be included. For clinical research, all histological measures, biochemical measures, and functional scores will be included.

### Risk of bias

Multiple tools will be used to assess the risk of bias for included studies. The Systematic Review Centre for Laboratory Animal Experimentation (SYRCLE) risk of bias tool will be applied to animal studies [35]. Ten domains will be addressed related to selection bias, performance bias, detection bias, attrition bias, reporting bias, and other biases. The Risk of Bias in Non-randomized Studies of Interventions (ROBINS-I) tool will be used to assess observational and quasi-randomized studies [36]. Seven domains will be used to assess risk including: confounding, participant selection bias, classification bias, deviation bias, bias due to missing data, outcome measurement bias, and bias in selection of reported results. Studies will be judged to have no information, or a low, moderate, serious, or critical risk of bias. For randomized controlled trials, the Risk of Bias 2 (RoB 2) tool will be used to establish the risk of bias [37]. Five domains including biases arising from the randomization process, from deviations from intended interventions, from missing outcome data, in measurement of the outcome, and in selection of the reported result will be analyzed. Overall risk of bias will be determined as low, some concerns, or high. All included studies will be independently scored by two reviewers, and consensus reached by discussion.

### Data synthesis and meta-analysis

Data synthesis and meta-analysis will be performed separately for preclinical and clinical studies, following published guidelines [37]. Given the sparsity of homogenous research, a qualitative analysis of common outcome variables will be conducted. Subgroups chosen for analysis will include tissue and/or MSCs derived from amniotic/chorionic membrane, amniotic fluid, umbilical cords, and/or umbilical cord-derived Wharton’s jelly versus experimental therapy and/or control. Results of meta-analyses extracted data will be summarized in tables and narrative interpretation provided, with emphasis on outcome measures.

## Discussion

The results of this review will be published in relevant peer reviewed scientific journals and presented at national or international conferences (‘Publications’) by the Investigators. This topic collates the information available regarding allogenic perinatal tissues and their respective uses for orthopedic regenerative medicine. This systematic review will have a beneficial impact on the present state of knowledge regarding this topic as it will help other clinicians and researchers identify the current information available along with the supporting studies.

## Documenting protocol amendments

Protocol amendments and updates will be documented via PROSPERO online register. The nature of the changes made will be recorded, dated and accessible along with the most recent version within the record audit trail under the systematic review protocol registration.

## Data Availability

The results of this review will be published in a relevant scientific journal or presented at national or international conferences (‘Publications’) by the Investigators.

## Abbreviations

ACM: Amnion/chorion membrane
ADCs: Adipose-derived cells
ADTs: Adipose-derived tissues
AF: Amniotic fluid
BMTs: Bone marrow transplants
MSCs: Mesenchymal stromal/stem cells
NSAIDs: Non-steroidal anti-inflammatory drugs
PICOS: Population, intervention, comparison, outcome, study design
PRISMA: Preferred reporting items for systematic reviews and meta-analyses
PRP: Platelet-rich plasma
SVT: Stromal vascular fraction
SYRCLE: Systematic review centre for laboratory animal experimentation
RoB 2: Risk of bias 2
ROBINS-I: The risk of bias in non-randomized studies of interventions
UC: Umbilical cord
UC-WJ: Umbilical cord-derived Wharton’s jelly
